# Parental Survey on Spanish‑English Bilingualism in Neurotypical Development and Neurodevelopmental Disabilities in the United States

**DOI:** 10.1007/s41252-023-00325-6

**Published:** 2023-03-16

**Authors:** Laura del Hoyo Soriano, Jennifer Villarreal, Leonard Abbeduto

**Affiliations:** 1. MIND Institute, University of California Davis, 50Th Street, Sacramento, CA 2825, USA; 2 Department of Psychiatry and Behavioral Sciences, University of California, Davis Health, Sacramento, CA, USA; 3. Graduate Program in Human Development, University of California, Davis, CA, USA

**Keywords:** Bilingualism, Neurodevelopmental Disabilities, Neurotypical Development, Parental Perceptions, Community Support

## Abstract

**Objectives:**

The cognitive and social benefits of bilingualism for children, including those with neurodevelopmental disabilities (NDDs), have been documented. The present study was designed to characterize and compare English and Spanish use in Hispanic families with and without NDDs residing in the U.S. as well as to understand parental perceptions of their child’s bilingualism and of community and professional support.

**Methods:**

We conducted an online survey of 84 Spanish-speaking parents of 4- to 24-year-olds with (*n* = 44) and without NDDs (*n* = 40) who were born in and living in the U.S.

**Results:**

We found that bilingualism was a desired goal for 95% of our families. We also found, however, that 17.1% of parents of children with NDDs have raised them as monolinguals English-speakers, as they thought there were reasons for that, while all families from the NT group raised their children in both languages. In addition, nearly 40% of the NDD children only speak English, compared to a 5% in the NT group. Finally, parents of children with NDDs cite a lack of support for bilingualism in the community (47.6% do not feel supported, compared to a 7.9% in the NT group) and recommendation from professionals as major factors for not raising their children as bilingual.

**Conclusions:**

The results suggest a need to educate professionals from many disciplines about the benefits of bilingualism for children with NDDs and for implementation of inclusion policies that provide access to dual-language programs.

Half of the world’s population uses two or more languages in their daily lives (Grosjean, 2021). In the United States (U.S.), more than 67 million people speak a language other than English at home according to the 2018 American Community Survey (ACS) from the Census Bureau. English–Spanish bilinguals represent 61% of all bilinguals in the U.S., making Spanish the second most frequently spoken language in the country (Grosjean, 2021). Indeed, Spanish is an official second language in many U.S. states. According to the Census Bureau, there are more than 41 million people aged 5 or older in the U.S. who speak Spanish at home.

There is considerable evidence that neurotypical (NT) children have the capacity to learn and speak two (or more) languages and that there are social and cognitive benefits of early bilingualism (Perani & Abutalebi, 2015; Rosselli et al., 2014). For example, measures of bilingual children’s total language growth, calculated by adding vocabulary scores across two languages, are typically equal to or greater than measures of monolingual children’s growth (Hoff et al., 2012, 2014; Silvén et al., 2014). The timing of bilingual exposure also seems to have an impact on bilingual development. One the one hand, NT who are exposed to both languages before 3 years of age (i.e., *simultaneous* bilinguals) achieve language milestones at similar ages to monolingual children and demonstrate language-appropriate morphosyntactic development (Paradis et al., 2011). On the other hand, those NT children who are exposed to the second language after age 3 years (i.e., *sequential* bilinguals) lag behind their same-age monolingual peers in acquiring the same language (Abrahamsson & Hyltenstam, 2009).

Despite the large body of literature documenting the capacity of NT children to learn and speak multiple languages and the cognitive, social, and linguistic benefits of early bilingualism, many young dual language learners in the U.S. do not receive support for their emergent bilingualism in school and beyond. In fact, some research suggests that these children may even be discouraged from continuing to use or learn their parent’s native language if that is a minority language (defined as a language less valued by society, spoken by fewer people and/or not present or less evident in the media and public institutions).

Children with neurodevelopmental disabilities (NDDs), who are already facing developmental challenges, also face a lack of support for using and learning their family language. Indeed, many professionals (e.g., physicians, speech-language therapists, psychologists, behavioral specialists, and teachers) discourage bilingualism and encourage families to expose their children with NDDs solely to the majority, or dominant, language of the society (e.g., English in the U.S.) to promote language development and academic success (Ijalba, 2016; Kay-Raining Bird et al., 2012; Uljarević et al., 2016). However, recent studies focused on children with various NDDs show no adverse effects of multilingual exposure (Edgin et al., 2011; Katsarou & Andreou, 2019; Uljarević et al., 2016; Ward & Sanoudaki, 2021). If anything, positive effects on the development of their cognitive skills, as well as on their communication and social functioning, have been observed. For example, bilingual children with autism are more likely to vocalize and utilize gestures when communicating, compared to their monolingual peers (Valicenti-McDermott et al., 2013; Zhou et al., 2019).

Several studies of autistic individuals have shown either no negative effects or positive effects of bilingualism on children with NDD development. A study of autistic bilinguals, for example, found that although the number of English words produced was lower compared to autistic monolinguals, the number of words produced in both languages combined was significantly higher for the bilingual group (Petersen et al., 2012). Moreover, a recent study in 7-to- 12-year-old bilingual and monolingual autistic children using narrative samples found that bilingual children with autism outperformed their monolingual peers with autism in both the maturity of their narrative productions (Peristeri et al., 2020). A 2021 study of 103 autistic children and adolescents showed a clear benefit for various aspects of theory of mind (i.e., the ability to attribute thoughts, emotions, and beliefs to others who hold thoughts and feelings different than one’s own) and executive functioning (i.e., goal-directed planning and behavior) for bilingual autistic participants, compared with their monolingual peers (Peristeri et al., 2021). Current research also suggest that bilingualism may have a positive effect in set-shifting (Gonzalez-Barrero & Nadig, 2019), visual attention, and working memory skills (Peristeri et al., 2020) in autistic children.

Studies of children with NDDs besides autism have yielded similar results. A recent study (King et al., 2021) that investigated the associations between language experience and non-linguistic cognitive variables in relation to Spanish and English semantic abilities in bilingual children with NT and children with specific language impairment (SLI) (e.g., children with no hearing loss and average non-verbal ability who still present a language delay, compared to NT children) found similar results for both groups. In particular, processing speed was related to vocabulary depth in English and Spanish with no effects for the language experience variables (i.e., age of exposure to English, language input, and language output). Another study demonstrated that simultaneous bilinguals with Down syndrome (DS) exhibited the same ability to learn novel words as NT bilinguals matched on non-verbal mental age and monolinguals with DS (Cleave et al., 2014). Studies have also shown that bilingual children with DS do not differ in their receptive and productive language skills or their phonological awareness skills from monolingual children with DS (Katsarou & Andreou, 2019; Ward & Sanoudaki, 2021). In addition, bilingualism has been shown to be a powerful cognitive reserve delaying the onset of dementia by approximately 4 years in neurotypical adults (Perani & Abutalebi, 2015), which could have its benefits for individuals with DS as well given their high risk of developing early onset Alzheimer’s disease (del Hoyo Soriano et al., 2015; Krinsky-McHale & Silverman, 2013).

In summary, research on individuals with NDDs and NT individuals has yielded results that are consistent with the position that parents should be supported in providing bilingual input to their children. In addition, a lack of exposure to the family’s primary language during childhood might have negative consequences on the social–emotional development of the child and their sense of heritage (Chen & Padilla, 2019; Ramírez-Esparza & García-Sierra, 2014). For example, children who do not develop and maintain their home language may lose their ability to communicate with grandparents (or, in some cases, even with parents) and other family members. These children risk becoming estranged from their cultural and linguistic heritage. In contrast, “those who can communicate in their family’s native language are able to establish a strong cultural identity, to develop and sustain strong ties with their immediate and extended families and thrive in a global multilingual world” (Hanson & Espinosa, 2016 p.2). In addition, early relationships established between parents and children, and the ways in which language conveys cultural meaning within these relationships, are important for the social–emotional development of the child (Byers-Heinlein & Lew-Williams, 2013; Byers-Heinlein et al., 2017; Ijalba, 2016). Thus, there are compelling reasons to actively support young NDD dual language learners’ bilingualism.

Given that Spanish is the second most frequently spoken language in the U.S., we surveyed Spanish-speaking parents of preschoolers to young adults with and without NDDs who were born in the U.S. Addressing the issues around bilingualism is especially important for individuals with NDDS because they are more dependent throughout their lives on the support of parent and professionals relative to neuro-typical individuals. Therefore, misinformation and negative attitudes regarding bilingualism (i.e., children being discouraged from their linguistic heritage) has the potential for especially profound effects on those with NDDs.

The objectives of this survey-based study were to characterize and compare English and Spanish use in Hispanic families with and without NDDs residing in the U.S., as well as to understand parental perceptions of their child’s bilingualism and of community and professional support through an online anonymous survey. Our research questions were as follows: (1) What is the Spanish and English language usage of U.S.-residing Hispanic families who have children, adolescents, and young adults with and without NDDs? (2) What are parent perceptions of their children bilingualism? (3) What are parent perceptions of the community and professional support for their children bilingualism? (4) Are there differences between NT and NDD families in language usage, parental perceptions of bilingualism, and perceived community and professional support for bilingualism?

## Method

### Participants

A total of 84 participating parents who self-identified as Hispanic completed the survey, 40 of whom were parents of NT children, adolescents, or young adults, and 44 of whom were parents of children, adolescents, or young adults with NDDs, which included autism, attention-deficit/hyperactivity disorder (ADHD), DS, and fragile X syndrome (FXS).

### Procedure

Participants were recruited from a university online volunteer research registry, as well as through a lab database of participants from previous studies, and the mailing list of national advocacy and family support organizations. We also distributed information to local university programs working Spanish speaking families.

The following were the inclusion criteria: the participating parent must be (1) older than 18 years and must consent to participate; (2) reside in the U.S.; (3) indicate that his/her primary language is Spanish; and (4) have a son/daughter between 4 and 24 years of age who was born in the U.S. The age range was selected to increase chances of (1) including children old enough to have developed some verbal skills (4 years of age) and (2) are still residing in the parental household (24 years of age), thereby making parent report appropriate. Sociodemographic data of participating parents and their children as well as the clinical characteristics of children with NDDs are presented in Table 1.

In completing the survey, families had to select “yes” or “no” regarding the following statement: “I am 18 years of age or older AND my primary/native language is Spanish AND I reside in the U.S. AND I have a son/daughter between 4 and 24 years of age who was born in the U.S.” If that was selected, families had to choose between the following two options: “My son/daughter has been diagnosed with developmental or intellectual disabilities, such as autism, Down syndrome, Fragile X, ADHD, and/or other” or “My son/daughter has NOT been diagnosed with a developmental or intellectual disability.”

### Measures

An online survey was designed for this study and delivered via Qualtrics — web-based software that enables the creation of surveys and generation of reports of aggregated responses (Qualtrics, Provo, UT). The online survey questionnaire was completed by parents and took a mean of 20 min for parents of individuals with NDDs and 12 min for parents of NTs. The questionnaire was available only in Spanish and divided into the three blocks (described below). If a parent reported having more than one child, we asked them to focus on only one of their children when responding to the survey. The complete questionnaires are available as supplementary files.

#### Block 1: Sociodemographic Data

Questions addressing parents’ sociodemographic data were incorporated into the survey to determine parental age, gender, country of birth, and age when they moved from their home country to the U.S.; U.S. state of current residence; employment status (employed/unemployed); household income; number of adults living at home; and number of children living at home.

Questions concerning the child’s sociodemographic data were also included in the first block of the survey to determine child age and sex and for parents of children with NDDs, child’s diagnosis; the child’s age when diagnosed; and whether the child was currently receiving language or behavioral services.

#### Block 2: Use of Language/s

In the second block of survey questions, the focus was on the use of language/s in the family. In particular, the following topics were addressed: (1) language/s practices by the parent/child/and other members of the family; (2) child’s language preferences; and (3) language/s to which the child is exposed in and out of the home. See Table 2 for more details, although we focus only on a subset of the questions in this paper.

#### Block 3: Parental Opinions on Bilingualism and Perception of Community Support

In this block of survey questions, the focus was on parent opinions regarding bilingualism and their perception of professional and community support for their children’s bilingualism. The following broad topics were addressed regarding the children’s bilingualism: (1) parental experience, perspectives, and feelings; (2) strategies and resources used; (3) professional advice received; and (4) community support. The questions addressing these topics were designed to be answered (a) using 5-point a Likert scale, (b) selecting one from several options, or (c) providing text in an open-ended format. See Table 3 for details of the key questions from this block.

### Data Analyses

Descriptive analyses (frequencies or means and standard deviations) were calculated for the survey questions. In addition, inferential statistics were conducted to compare NT and NDD families on key sociodemographic variables, as well as for the major questions of interest regarding bilingualism. One-way ANOVAs were used to address group-differences regarding quantitative data, such as interval or ratio variables (e.g., age). Given the non-normality distribution of most of the dependent variables, non-parametric tests were used to address interactions as well as categorical variables. In particular, Pearson chi-square test was used to determine if there were non-random associations between each pair of categorical variables. Our sample size of 84 participants allowed us to find differences between data on the NDDs and NT group with an alpha of 0.05 and a power of 0.73. Note that on the two open-ended questions (see two last rows in Table 3) only descriptive analyses were performed. All statistical analyses were performed using the SPSS statistical software packages (Version 18.0; SPSS Inc., Chicago, IL, USA). G-power was used to compute sample size and power.

## Results

### Sociodemographic Data of Families with and Without NDDs

See Table 1 for these data. Chi-square tests showed differences between the NT and NDD groups, regarding the proportion of parents who were born inside and outside the U.S., such that a higher number of parents in the NDD group were born outside the U.S., *X*^2^ (1, *N* = 84) = 37.42, *p* < 0.001. An ANOVA showed no differences between the NT and NDD groups, regarding parental age when they moved to the U.S. (for those parents who had moved). There were differences in current parental age, such that parents from the NT group were significantly younger that those from the NDD group, *F* (1, *N* = 82) = 16.2, *p* < 0.001. Chi-square tests also showed gender differences across the NT and ND groups both in terms of parents *X*^2^ (1, *N* = 84) = 22.39, *p* < 0.001, and offspring *X*^2^ (1, *N* = 83) = 6.29, *p* = 0.01, seen in Table 1. No significant group differences were observed in terms of parent employment status, household income, number of adults/children living at home, or child age.

### Use of Language in Families with and Without NDDs

As seen in Table 2, all parents who participated in this survey considered themselves native Spanish speakers and were able to communicate in English to some extent. Most of the parents from the NT group (i.e., 90%, *n* = 36) considered themselves to be fluent in both English and Spanish; however, a considerably lower number of parents from the NDD group (i.e., 29.5%, *n* = 13) considered themselves to be fluent in both languages, *X*^2^ (1, *N* = 79) = 40.49, *p* < 0.001. Regarding the language/s that the parent uses to communicate with their child, there were group differences *X*^2^ (1, *N* = 83) = 6.87, *p* = 0.03. In particular, 25% of the parents from the NT group (*n* = 10) communicate with their child only in Spanish and 70% (*n* = 28) communicate in both languages. In contrast, parents in the NT group rarely use only English (5%, *n* = 2). The parents of the NDD children were more likely to use only Spanish (44.1%, *n* = 19) or only English (14%, *n* = 6), and less likely to use both languages (41.9%, *n* = 18) relative to the NT group (see Table 2). Nearly all the parents (95%, *n* = 38) from the NT group reported that their child was able to understand and speak both English and Spanish. Although 88.6% (*n* = 39) parents from the NDD group reported that their child was able to understand both English and Spanish, only 52.3% of the parents (*n* = 23) reported that their children with NDD communicate in both languages, *X*^2^ (1, *N* = 80) = 14.05, *p* < 0.001. Note, however, that 9.1% of the children with NDDs were non-verbal. In addition, 68.4% of the parents from the NT group and 82.6% of the parents from the NDD group stated that their bilingual child used more than one language in the same utterance, with no significant group differences (*X*^2^ (1, *N* = 61) = 1.49, *p* = 0.18). Interestingly, most of the children in both groups were exposed to both languages in (97.5% NT; 75.6% NDDs) and out (85% NT; 75.6% NDDs) of the home. Finally, nearly all the parents from the NT group (92.3%) believed their children understood and spoke English as well as or better than their peers, whereas only 47.5% of the parents of verbal children from the NDD group thought that their children understood/spoke English as well as or better than their peers (see Table 2). With regard to Spanish, 70% of the parents from the NT group believed their children understood and spoke Spanish as well as or better than their peers, whereas only 28.6% of verbal children from the NDD group thought that their children understood and spoke Spanish as well as or better than their peers (see Table 2 for details).

### Parental Perceptions of Bilingualism in Families with and Without NDDs

As seen in Table 3, less than 5% of the parents from both groups stated that it was not at all important to them for their child to become bilingual. For those parents for whom it was important to some extent for their child to become bilingual, the reasons differed for the two groups of parents. For the parents of NT children, the most important reason to be bilingual was that “It gives more job opportunities in life,” followed by “To maintain our Hispanic culture.” In contrast, for the parents of children with NDDs, the most important reason to be bilingual was to “Maintain our Hispanic culture,” followed by “To communicate with family members who do not speak English.”

Interestingly, when asked if they had ever believed that there were reasons for their child to not be bilingual, one-third (33.3%) of the parents from the NDD group said “yes,” compared to only one parent (2.5%) from the NT group who believed this, which was a significant difference, *X*^2^ (1, *N* = 82) = 13.03, *p* < 0.001. Among the parents of the NDD individuals, the most common reason (35.7%) stated for ever believing that their child should not be bilingual was “Due to his diagnosis.” When asked for their current views on their child being bilingual, about 24.4% of parents from the NDD group indicated that there were reasons to not be bilingual, whereas no parent from the NT group believed this, *X*^2^ (1, *N* = 81) = 11.13, p < 0.001. Among the parents from the NDD group, the most common reason for this belief was “Due to language difficulties” (30%) followed by “Due to his diagnosis” (20%). Whereas all parents from the NT group had decided to raise their children as bilingual, fewer parents (82.9%) from the NDD group had decided this, *X*^2^ (1, *N* = 81) = 7.48, *p* = 0.006. Of the 34 parents from the NDD group who decided to raise their child as a bilingual, only 1 regretted the decision, stating that “Because sometimes other children make jokes on his accent.” Interestingly, of the 7 parents from the NDD group who decided to raise their child as monolingual, 5 regretted that decision, stating reasons such as “The child cannot communicate with his loved ones,” “I see other children with the same condition who speak both languages so maybe he could have done the same,” “Now is too late,” “It would have given him more opportunities in life.” In indicating why they had raised their children as monolingual, parental comments included the following: “Lack of access to services,” “Too difficult,” “A second language will confuse the child,” and “Just followed a professional recommendation.”

### Parental Perceptions of Professional and Community Support in Families with and Without NDDs

As seen in Table 3, of those parents from the NT group who received a recommendation from a professional (e.g., primary care physicians (PCP), speech language pathologists (SLP), psychologists, and teachers), none was ever told to avoid raising their children as bilingual in comparison to the parents from the NDD group, some of whom were told by various professionals to raise their children only in English (e.g., 17.1% PCP, 19.5% SLP, 12.2% psychologists, 7.3% social workers, 12.2% behavior specialists, and 12.2% teachers). See Table 3 for details. In addition to that, nearly all parents (92.1%) from the NT group felt supported by the community in raising their child with more than one language, whereas only a little more than half the parents (52.4%) in the NDD group felt supported, *X*^2^ (1, *N* = 80) = 15.37, *p* < 0.001. At the same time, however, most parents (97.4% NT; 90.2% NDD) from both groups believed that bilingualism is viewed positively in their communities, with no group differences, in this regard NT group, *X*^2^ (1, *N* = 80) = 1.76, *p* = 0.2. In contrast, 27.9% of the parents from the NDD group reported that their child had indicated not wanting to learn or speak Spanish, which was a situation rarely reported (2.6%) by the parents from the NT group, *X*^2^ (1, *N* = 82) = 9.85, *p* = 0.002. The most commonly reported resource to support their children’s bilingualism were bilingual schools for both groups (60% NT; 42.9% NDD). Parents from the NDD group also mentioned “public libraries,” “TV in Spanish,” and “language therapy” to be useful resources. Finally, to the open-ended question “What advice (if any) would you give to other parents who want to raise their children as bilinguals?” the most repeated advice from the parents in the NDD group was “talk to your children in Spanish,” followed by “be patient and consistent,” and “start from early childhood.” The most repeated advice in the NT group was “watch TV in Spanish” followed by “talk to your child in Spanish,” and then “be persistent”.

## Discussion

There is extensive research on bilingualism in NT children from multiple perspectives — linguistic, cognitive, biological, social, educational, and more — with consistent evidence of a range of benefits for development and no adverse consequences of learning and use more than one language. Although there is a much smaller literature on bilingualism in individuals with NDDs, the emerging data also suggest benefits rather than adverse consequences. More studies on this topic in NDD populations, however, are needed to provide timely, appropriate, and effective support and intervention to help those with NDDs become bilingual, especially in multicultural and multilingual countries such as the U.S. in which bilingualism should be a right for these families and not a privilege.

This survey is a necessary contribution, as for the first time, the perception of native Spanish speaking parents of children with and without NDDs residing in the U.S. regarding their child’s bilingualism as well as their perception of the community and professional support they receive for bilingualism is explored. Understanding families’ perspectives is essential to building policies to provide them the support needed for their children to develop their skills in their home language/s as well as in English. To this end, we surveyed Spanish-speaking parents of 4- to 24-year-olds with and without NDDs who were born and residing in the U.S. with objective being to characterize and compare the use of English and Spanish in Hispanic families, as well as learn parental perceptions of their children’s bilingualism and professional and community support.

Before discussing the findings, it is important to acknowledge the group-related differences in several demographic factors. For example, more parents from the NDD group were born outside of the U.S. and considered themselves not to be fluent English speakers, compared to the parents in the NT group. Such differences could have impacted the pattern of results for the language preferences and community characteristics. At the same time, however, both groups of parents maintained Spanish as their primary language, and this factor would reasonably be expected to be a driver of parental preferences regarding their children’s bilingualism. Nonetheless, our findings should be seen as preliminary and as suggesting hypotheses for future research with larger, matched participant samples.

Our first research objective was to characterize and compare Spanish and English language usage of U.S.-residing Hispanic families who have children, adolescents, and young adults with and without NDDs. In this regard, our results show that virtually all NT and NDD children, adolescents, and young adults can understand both English and Spanish but fewer NDD people use Spanish in their speech, compared to NT. In addition, it is reported that both NT and NDDs tend to prefer English over Spanish or no preference at all.

Importantly, most of the families who raised their children as bilinguals reported that both parents and children use English and Spanish words in the same sentence, with no significant differences between groups in this regard. Given the language flexibility reported by parents, we suggest that language assessments, whether for clinical purposes or research, should carefully consider family preferences and practices when deciding on the language/s of assessment, as well as when interpreting the results of assessments. That is, counting language production in both languages when evaluating the language skills of a child regardless of the language in which the assessment has been administered. More generally, it will be increasingly necessary to take a more dynamic approach to language assessments using language practices of bilingual families as the norm in terms of both administration and interpretation. This is in line with the concept of “translanguaging” (Otheguy et al., 2015), described as the process whereby multilingual speakers use their languages as an integrated communication system. In fact, there is evidence that the use of expressive language sampling procedures involving parent–child interaction elicit numerous language shifts in talk by Spanish–English bilingual parents and their children with autism (del Hoyo Sorianoet al., 2021a, b).

Adopting a more dynamic approach when evaluating bilingual children would entail, among other things, allowing the child to shift between languages when speaking and to interact with a bilingual examiner who can also match the child’s within-sample language shifts so that the child feels comfortable and fully supported, and of course consider language production of both languages when interpreting the assessment results. This is especially important as a correct interpretation of results from such assessment may have a direct impact in professional recommendations regarding bilingual practices.

Finally, most of the families in the present study indicated that their children were exposed to both English and Spanish both in and outside the home. This is interesting as previous research suggests that it is of critical importance to ensure a high amount of exposure to a language for language growth to take place (Kay-Raining Bird et al., 2016). Therefore, our results together indicate that efforts should be made to facilitate the exposure of the weaker language through services available to these children as early as possible and to educate health care and education professionals by creating evidence-based guidelines for the best practices to support early bilingualism in NDD populations.

It is important to note, however, that although both groups are exposed to Spanish inside and outside the house, we still see that NT children are more likely to speak Spanish (or master it) and to be raised as bilinguals, compared to NDDs. Therefore, even though we see similar patterns of language exposure in families with and without NDDs, it seems like NDD children are less likely to speak Spanish, probably given a lack of resources specific for these populations. Indeed, the reason cited by all parents who decided to raise their children as monolingual was a lack of access to services, suggesting that NDD families might have chosen to raise their children as bilingual if they had the resources available to them to do it. These results are in line with the second research objective concerning parental perceptions on their children’s bilingualism.

Furthermore, we only see that those with NDDs (but not NT) have ever thought there are reasons for their child not to become bilingual as they thought they would not be capable for a variety of reasons (e.g., diagnosis and language difficulties). One explanation behind such thoughts could be the sociodemographic difference between parents regarding their English language skills; however, a contributing factor might also be the lack of resources available in the community to support bilingualism in children with atypical development, as well as less encouraging professionals. Having this information in mind could help in the understanding of the societal changes needed to help bilingual Hispanic families of children with NDDs maintain their linguistic heritage and accrue the benefits of bilingualism.

Concerning parental perceptions of professional and community support, our results are in line with previous studies of autism in showing that parents of children with NDD were often told by professionals to speak only the majority language to their children or they chose to do so themselves because they feared that exposure to two languages would cause or exacerbate developmental challenges or because they simply could not access services in their native language (Ijalba, 2016; Jegatheesan, 2011; Kay-Raining Bird et al., 2012; Yu, 2013). Importantly, in these studies parents expressed personal loss and sadness if they chose to speak only English to their autistic children.

A number of parents also expressed discomfort and difficulty when speaking a non-native language with their child (Yu, 2013) or said they talked less frequently to their child when they used the majority language because it felt less natural. Our results are in line with previous studies as suggested that even if Spanish is the primary language of the parents, some families still choose to use only English to communicate with their children with the goal of not hampering their language development, with this being more common in families of NDD children. However, the vast majority of parents, regardless of whether their children are NT or NDD, value bilingualism, and most of the parents who decided to raise their children as monolinguals indicated that regret with that decision, which is in line with previous research (e.g., Yu, 2013).

### Limitations and Future Research

Although the current study makes a necessary contribution by detailing the experiences of Hispanic families of children, adolescents, and young adults with and without NDDs residing in the U.S., some limitations must be acknowledged. First, despite the desirability to recruit a sample with similar sociodemographic characteristics in both NT and NDD groups, we can observe important differences between groups such as the country of birth of the parent. Mean age and sex frequencies of the parents and the children are also diverse between groups, all of which should be considered as possible confounders when interpreting our findings.

Second, it is important to recognize that our sample size is relatively small for a survey-based study, and we are dealing with several missing data in some of the questions.

Third, we did not include a question for those families who decided to raise their children as bilinguals, which was asking whether it was a simultaneous (from birth or soon after) or sequential exposure/learning (usually after the 3 years of age). Such questions would have provided information on whether bilingual families from each group choose one over the other pattern of exposure, as well as for those sequential bilinguals, whether the first language introduced was English or Spanish. This is an important topic given that Spanish was the primary language for all the parents in the present study and all of them reported that their children were exposed to Spanish at home (directly or indirectly). Thus, it could be that some of our bilingual families might have naturally first introduced Spanish to their child, then introduced English with school or the other way around.

In addition, we did not collect information regarding birth order from those parents with more than one child (e.g., first-born children vs their later-born siblings); this is a limitation as the position a child occupies among their siblings may be relevant (e.g., language experience of a child with older siblings, compared to younger).

Finally, we must acknowledge limitations associated with online survey-based studies, such as respondent self-selection (i.e., the decision to participate in the survey is left entirely up to individuals which gives rise to research bias in terms of motivation/demographics) and accessibility issues (i.e., access to a computer, tablet or phone) and internet).

Future studies with larger data sets, including families for whom primary language is other than Spanish, and including questions such as siblings birth order, pattern, and timing of language exposure should be conducted to extend current results. In addition, non-survey-based studies on the topic need to be performed to support our data.

## Supplementary Material

Suplementary 1

Suplementary 2

## Figures and Tables

**Table 1:** Demographic data of participants (*n* = 84)

	NT group (*n* = 40)	*n*	NDD group (*n* = 44)	*n*
Age of parent	*M* = 37.24; *SD* = 6.038; (26–50)	38	*M* = 42.3; *SD* = 7.5; (28–62)	44
Sex of parent	21- Female (52.5%)	40	42- Female (95.5%)	44
	19- Male (47.5%)		2- Male (4.5%)	
Country of birth of parent	31- USA (77.5%)	40	5- USA (11.6%)	43
	1- Argentina (2.5%)		2- El Salvador (4.7%)	
	1- Colombia (2.5%)		1- Spain (2.3%)	
	1- Ecuador (2.5%)		2- Guatemala (4.7%)	
	1- Spain (2.5%)		1- Jamaica (2.3%)	
	5- Mexico (12.5%)		1- Peru (2.3%)	
			1- Dominican Republic (2.3%)	
			30- Mexico (69.8%)	
If born outside the USA, age of the parent when moved	*M* = 25; *SD* = 8.15; (15–38)	8	*M* = 22.8; *SD* = 5.6; (12–38)	39
Employment status	27- Employed (67.5%)	40	25- Employed (56.8%)	44
	13- Unemployed (32.5%)		19- Unemployed (43.2%)	
Household income	1- Less than $10,000 (2.5%)	40	3- Less than $10,000 (6.8%)	44
	4- $20,000–$29,999 (10%)		1- $10,000–$19,999 (2.3%)	
	5- $30,000–$39,999 (12.5%)		2- $20,000–$29,999 (4.5%)	
	6- $40,000–$49,999 (15%)		5- $30,000–$39,999 (11.4%)	
	7- $50,000–$59,999 (17.5%)		5- $40,000–$49,999 (11.4%)	
	6- $60,000–$69,999 (15%)		8- $50,000–$59,999 (18.2%)	
	3- $70,000–$79,999 (7.5%)		3- $60,000–$69,999 (6.8%)	
	2- $80,000–$89,999 (5%)		1- $80,000–$89,999 (2.3%)	
	2- $100,000–$149,999 (5%)		2- $90,000–$99,999 (4.5%)	
	2- $150.000–$199,999 (5%)		3- $ 300.000 or more (4.5%)	
	2- I prefer not to answer (5%)		3- I don’t know (6.8%)	
			9- I prefer not to answer (20.5%)	
Number of adults living at home	*M* = 2.6; *SD* = 0.955; (2–5)	40	*M* = 2.8; *SD* = 1; (2–5)	43
Number of children living at home	*M* = 2.9; *SD* = 0.841; (0–4)	40	*M* = 2; *SD* = 1.1; (0–5)	43
Age of child	*M* = 9.8; *SD* = 3.84; (4–18)	40	*M* = 11.7; *SD* = 5.4; (4–25)	40
Sex of child	18- males (45%)	40	31- males (72.1%)	43
	22- females (55%)		12- females (27.9%)	
Only for participants with NDDs				
Diagnosis received			29- Autism Only (65.9%)	44
			6- Autism and ADHD (13.6%)	
			2- ADHD only (4.5%)	
			1- DS and Autism (2.3%)	
			2- FXS and Autism (4.5%)	
			1- FXS only (2.3%)	
			3- Non-specific ID (4.5%)	
Age when diagnosed			*M* = 5; *SD* = 3.2 (2–11)	25
Currently under behavioral and/or language therapy			42- Yes (95.5%)	44
			2- No (0.5%)	

Note that missing values were due to parent not completing such question on the survey

**Table 2:** Use of language/s (English and Spanish) by participating families (*n* = 84)

	NT group (*n* = 40)	*n*	NDD group (*n* = 44)	*n*
Language/s that parent speaks fluently	4- Only Spanish (10%) 36- English and Spanish (90%)	40	31- Only Spanish (71.5%) 13- English and Spanish (29.5%)	44
Language/s parent uses to communicate with child	10- Only Spanish (25%) 2- Only English (5%) 28- Spanish and English (70%)	40	19- Only Spanish (44.1%) 6- Only English (14%) 18- Spanish and English (41.9%)	43
Parent or other adult at home uses more than one language in the same utterance	26- Yes (65%) 14- No (35%)	40	23- Yes (56.1%) 18- No (43.9%)	41
Language/s child understands	38- English and Spanish (95%) 2- Only English (5%)	40	39- English and Spanish (88.6%) 5- Only English (11.4%)	44
Language/s child speaks	38- English and Spanish (95%) 2- Only English (5%)	40	23- English and Spanish (52.3%) 17- Only English (38.6%) 4- Non-verbal (9.1%)	44
If speaks more than one, child preference for one language	18- Prefers English (47.4%) 15- No preference (39.5%) 5- Prefers Spanish (13.2%)	38	10- Prefers English (45.5%) 7- No preference (31.8%) 5- Prefers Spanish (22.7%)	22
Does the child use more than one language in the same utterance	26- Yes (68.4%) 12- No (31.6%)	38	19- Yes (82.6%) 4- No (17.4%)	23
Number of languages the child is exposed inside the house	39- English and Spanish (97.5%) 1- Only Spanish (2.5%)	40	33- English and Spanish (75.6%) 8- Only Spanish (19.5%)	41
Number of languages the child is exposed outside the house	34- English and Spanish (85%) 6- Only English (15%)	40	31- English and Spanish (75.6%) 7- Only English (17.1%)	41
Who talks to the child in Spanish outside home? (Select all that apply)	29- Friends and relatives (82.9%) 16- Teachers (45.7%)	35	24- Friends and relatives (66.7%) 4- Therapists and doctors (11.1%) 8- School (22.2%)	36
Where is the child exposed to Spanish outside the house? (Select all that apply)	34- Leisure activities (97.1%) 12- School (34.3%)	35	24- Leisure activities (66.7%) 8- School (22.2%) 4- Therapy settings (11.1%)	36
Who talks to the child in English outside home? (Select all that apply)	40- Friends and relatives (100%) 35- Teachers (87.5%)	40	26- Friends and relatives (63.4%) 26- Teachers (63.4%) 15- Therapists and doctors (36.6%)	41
Where is the child exposed to English outside the house? (Select all that apply)	15- Leisure activities (37.5%) 40- School (100%)	40	18- Leisure activities (43.9%) 41- School (100%) 9- Healthcare settings (21.9%)	41
How well parent thinks child understand/talks English and/or Spanish compared to same age peers?	--------English Understands: 36- Same or better than peers (92.3%) 3- Worse than peers (7.7%) Talks: 36- Same or better than peers (92.3%) 3- Worse than peers (7.7%) --------Spanish Understands: 28- Same or better than peers (70%) 12- Worse than peers (30%) Talks: 27-Same or better than peers (67.5%) 13- Worse than peers (25%)	39 39 40 40	--------English Understands: 19- Same or better than peers (47.5%) 21- Worse than peers (52.5%) Talks: 18- Same or better than peers (45%) 18- Worse than peers (45%) 4-Does not talk at all (10%) --------Spanish Understands: 15- Same or better than peers (28.6%) 23- Worse than peers (54.8%) 3- Does not understand at all (7.2%) Talks: 10- Same or better than peers (23.3%) 21- Worse than peers (48.8%) 11- Doesn’t talk at all (25.6%)	40 40 42 43

Note that missing values were due to parent not completing such question on the survey and/or Spanish compared to same age peers?

**Table 3:** Parental opinions on bilingualism and perception of community support (*n*=83)

	NT group (*n* = 40)	*n*	NDD group (*n* = 45)	*n*
How important is for the parent for his/her child to become bilingual	11- Extremely important (27.5%) 17- Very important (42.5%) 7- Important (17.5%) 4- Somewhat important (10%) 1- Not important at all (2.5%)	40	22- Extremely important (51.2%) 15- Very important (34.9%) 1- Important (2.3%) 3- Somehow important (7%) 2- Not important at all (4.7%)	43
If other than “Not important at all:” The most important reason why you think it is important	27- It gives more opportunities in life/job (69.2%) 7- To maintain our Hispanic culture (18%) 3- Because we live in a bilingual country/community (7.6%) 2- To communicate with family members who do not speak English (5.1%)	39	17- To maintain our Hispanic culture (41.5%) 12- To communicate with family members who do not speak English (29.3%) 5- Because we live in a bilingual country/community (12.2%) 7- It gives more opportunities in life/job (17.1%)	41
Ever think there were reasons for child not to become bilingual	1- Yes (2.5%) 39- No (97.5%)	40	14- Yes (33.3%) 28- No (66.7%)	42
If yes, which ones (open label)	1- It was hard for him to talk in Spanish (2.5%)	1	5- Due to their diagnosis (35.7%) 1- Don’t feel supported by the community (7.1%) 1- Community resources are only in English (7.1%) 1- Recommended by their primary School (7.1%) 1- It’s very difficult for them and we will confuse him (7.1%)	14
Does the parent think that there are reasons for his/her child not to become bilingual nowadays	0- Yes (0%) 40- No (100%)	40	10- Yes (24.4%) 31- No (75.6%)	41
If yes; which ones (open label)		0	3- Language difficulties (30%) 2- Due to his diagnosis (20%) 2- It’s hard for him (10%) 1- He gets frustrated (10%)	10
Did parent decide to raise his/her child as a bilingual/monolingual	0- Monolingual (0%) 40 – Bilingual (100%)	40	7- Monolingual (English) (17.1%) 34- Bilingual (82.9%)	41
Does parent regret the decision made?	40- No 0- Yes	40	7- Monolingual (1 no, 5 yes) 34- Bilingual (33 no, 1 yes)	41
If bilingual: (1) Strategies parents used or are using to support their child bilingualism (select all that apply)	19- My child receives or has received classes to support his/her second language (47.5%) 13- Talk both languages at home (32.5%) 35- Reads books in both languages (87.5%) 35- Watches TV in both languages (87.5%)	40	9- My child receives or has received classes to support his/her second language (26.5%) 1- My child receives services from a SLP (2.9%) 17- Talk both languages at home (50%) 16- Reads books in both languages (47.1%) 23- Watches TV in both languages (67.6%)	34
How parents qualify their success in helping their children to become bilingual	2- Not successful at all (5%) 6- Somewhat successful (15%) 23- Successful (57.5%) 5- Very successful (12.5%) 4- Extremely successful (10%)	40	2- Not successful at all (5.8%) 20- Somewhat successful (58.8%) 6- Successful (17.6%) 2- Very successful (5.8%) 4- Extremely successful (11.8%)	34
What parents think has worked the most to support their child bilingualism (open-ended question)	14- Watching TV in both languages (38.9%) 11- Reading in both languages (30.6%) 5- Talking to my child only in Spanish (13.9%) 5-Talking both languages at home (13.9%) 1- Bilingual school (2.8%)	36	14- Talk in Spanish at home 2- Listen to songs in Spanish 2- Watch TV in Spanish 4- Read in Spanish 3- Be patient and supportive 2- Going to a bilingual school	27
If monolingual: reasons behind the decision (select all that apply)		0	7- I don’t have access to services to help my child become bilingual (100%) 4- I think it will be too difficult for my child (57.1%) 3- I’m afraid I would confuse my child (42.9%) 2- A professional told me to raise my child in one language (28.6%) 1- My family/friends would not approve raising my child as a bilingual (14.3%)	7
Ever told by a professional not to raise their children as bilinguals	yes (0%)	40	10 yes (24.4%)	41
Opinions/recommendations (if any) parents have received from professionals regarding raising or not their children as bilinguals	PCP: 7- raise your child as bilingual (17.5%) Speech language Pathologist: 4- raise your child as bilingual (10%) Psychologists 3- raise your child as bilingual (7.5%) Social workers 4- raise your child as bilingual (10%) Behavioral specialists 4- raise your child as bilingual (10%) Teachers 5- raise your child as bilingual (12.5%)	40	PCP: 7- don’t raise your child as bilingual (17.1%) 9- raise him/her as bilingual (22%) Speech language Pathologist: 8- don’t raise bilingual (19.5%) 10- raise as bilingual (24.4%) Psychologists 5- don’t raise (12.2%) 9- raise as bilingual (22%) Social workers 3- don’t raise (7.3%) 6- raise as bilingual (14.6%) Behavioral specialists 5- don’t raise (12.2%) 10- raise as bilingual (24.4%) Teachers: 5- don’t raise (12.2%) 11- raise bilingual (26.8%)	41
Do parents feel supported by the community to raise their child in more than one language?	35- Yes (92.1%) 3- No (7.9%)	38	22- Yes (52.4%) 20- No (47.6%)	42
Do parents think that bilingualism is considered something good in their community?	38-Yes (97.4%) 1- No (2.6%)	39	37- Yes (90.2%) 4- No (9.8%)	41
Child ever told them that he/she doesn’t want to learn or speak Spanish?	0- Yes (2.6%) 38- No (97.4%)	39	12- Yes (27.9%) 31- No (72.1%)	43
What resources (if any) would they recommend to other parents who want to raise their children as bilinguals (open label)	3- Bilingual schools (60%) 1- Bilingual Books (20%) 1- Spanish TV (20%)	5	6- Bilingual Schools (42.9%) 3- Go to libraries (21.4%) 2- Watch TV Spanish (14.3%) 3- Language therapy (21.4%)	14
What advice (if any) they would give to other parents who want to raise their children as bilinguals (open label)	8- Watch TV in Spanish (47.1%) 5- Talk in Spanish (29.4%) 3- Be persistent (17.6%) 1- Read in Spanish (5.8%) 1- Visit your home country (2.5%)	17	15- Talk in Spanish (46.9%) 10- Be patient/constant (31.3%) 5- Start in early childhood (15.6%) 2- Go to bilingual school (6.3%)	32
				

Note that missing values were due to parent not completing such question on the survey

## Data Availability

All the data are available at the Open Science Framework (https://osf.io/2puwz/).
