# Supplementary material for: Parental Survey on Spanish‑English Bilingualism in Neurotypical Development and Neurodevelopmental Disabilities in the United States
Source: Adv Neurodev Disord. Author manuscript; Available in PMC 2023 Dec 1. (PMC10664973; doi:10.1007/s41252-023-00325-6)
Supplement: Suplementary 1 [file NIHMS1895179-supplement-Suplementary_1.pdf]

SEBNCUS Grant: Survey - Neurotypical group

Skip To: End of Survey If Usted ha sido redirigido al final de la encuesta porque ha seleccionado: "No, he leído y comprend... Is Displayed

Display This Question:

If Si a continuación selecciona la opción "Sí, doy mi consentimiento", afirmará que: Ha leído la inf... = Sí, he leído y comprendido la información anterior y doy mi consentimiento. A continuación, escribe tu nombre y apellidos conforme consientes participar:

Q3.4 Para comenzar, nos gustaría obtener información sobre usted y su hogar.

¿En qué país nació usted?

- ◯ En los Estados Unidos (1)
- ◯ Otro pais, especifique cual: (2) ____

Display This Question:

If Para comenzar, nos gustaría obtener información sobre usted y su hogar. ¿En qué país nació usted... = Otro pais, especifique cual:

Q3.5 ¿Qué edad tenía usted cuando se mudó de su pais de origen a los EE. UU.?

____

Display This Question:

If If ¿Qué edad tenía usted cuando se mudó de su pais de origen a los EE. UU.? Text Response Is Displayed

Q3.6 ¿Cuál fue el nivel más alto de educación que usted completó en su país de origen?

- ◯ Escuela infantil - educación primaria (iniciada o completada) (1)
- ◯ Educación secundaria - Iniciada pero no completada (2)
- ◯ Graduado escolar: Bachillerato (3)
- ◯ Universidad o escuela técnica - Iniciada pero no completada (4)
- ◯ Graduado: Título universitario técnico (5)
- ◯ Graduado: Licenciatura (6)
- ◯ Escuela de posgrado - Iniciada pero no completada (7)
- ◯ Graduado: Maestría / doctorado / otro título de posgrado (8)

Skip To: Q3.7 If ¿Cuál fue el nivel más alto de educación que usted completó en su país de origen? , Escuela infantil - educación primaria (iniciada o completada) Is Displayed

---

Display This Question:

If Para comenzar, nos gustaría obtener información sobre usted y su hogar. ¿En qué país nació usted... = Otro pais, especifique cual:

Q3.7 ¿Estuvo empleado durante los últimos 2 años antes de mudarse de su país de origen?

- ◯ Sí (1)
- ◯ No (2)

Display This Question:

If ¿Estuvo empleado durante los últimos 2 años antes de mudarse de su país de origen? = Sí

Q3.8 ¿Cuántas horas trabajó en promedio cada semana?

- ◯ 30 horas semanales o menos (1)
- ◯ 31 horas semanales o más (2)

Skip To: Q3.9 If ¿Cuántas horas trabajó en promedio cada semana? , 30 horas semanales o menos Is Displayed

Display This Question:

If Para comenzar, nos gustaría obtener información sobre usted y su hogar. ¿En qué país nació usted... = En los Estados Unidos

Q3.9 ¿En qué estado de EE. UU. reside actualmente usted y su familia?

____

Q3.10 ¿Cuántos adultos viven en su hogar incluyéndose a usted?

____

Q3.11 ¿Cuántos niños viven en su hogar?

____

Q3.12 ¿Qué edad tiene usted? (en años):

____

Q3.13 Seleccione con que género se identifica usted:

- ◯ Mujer (1)
- ◯ Hombre (2)
- ◯ Género no binario (3)
- ◯ Otro (especifique) (4) ____
- ◯ Prefiero no contestar (5)

Q3.14 Seleccione con que origen étnico se identifica usted:

- ○ Hispano o Latino (1)
- ○ No Hispano o Latino (2)
- ○ Otro (especifique) (3) ____
- ○ Prefiero no contestar (4)

Q3.15 ¿Está usted empleado/a actualmente?

- ○ Sí (1)
- ○ No (2)

Display This Question:

If ¿Está usted empleado/a actualmente? = Sí

Q3.16 ¿Cuántas horas por semana trabaja usted actualmente (en promedio)?

- ○ 30 horas semanales o menos (1)
- ○ 31 horas semanales o más (2)

Q3.17 ¿Antes de la pandemia de COVID-19 usted trabajaba en casa, fuera de casa o no trabajaba?

- ○ En casa (1)
- ○ Fuera de casa (2)
- ○ Antes de la pandemia no estaba empleado/a (3)

Q3.18 ¿Ha experimentado usted algún cambio en su situación laboral debido a la pandemia de COVID-19 (puede elegir más de una opción si así lo considera)?

- ○ No (1)
- ○ Sí, aumento de horas (2)
- ○ Sí, horario reducido (3)
- ○ Sí, perdí mi empleo (4)
- ○ Sí, tuve que dejar mi trabajo para cuidar de mi familia (5)
- ○ Sí, comencé a trabajar principalmente desde casa (6)

Display This Question:

If Para comenzar, nos gustaría obtener información sobre usted y su hogar. ¿En qué país nació usted... = Otro pais, especifique cual:

Q3.19 ¿Continuó usted sus estudios después de mudarse a los EE. UU.?

- ○ No (1)
- ○ Sí (2)

Display This Question:

If Para comenzar, nos gustaría obtener información sobre usted y su hogar. ¿En qué país nació usted... , Otro pais, especifique cual: Is Displayed

And ¿Continuó usted sus estudios después de mudarse a los EE. UU.? = Sí

Q3.20 ¿Cuál es el nivel más alto de educación que usted ha completado en los EE. UU.?

- ○ Escuela infantil - educación primaria (iniciada o completada) (1)
- ○ Educación secundaria - Iniciada pero no completada (2)
- ○ Graduado escolar: Bachillerato (3)
- ○ Universidad o escuela técnica - Iniciada pero no completada (4)
- ○ Graduado: Título universitario técnico (5)
- ○ Graduado: Licenciatura (B.A./B.S.) (6)
- ○ Escuela de posgrado - Iniciada pero no completada (7)
- ○ Graduado: Maestría / doctorado / otro título de posgrado (8)

Q3.21 ¿Cuál es el ingreso bruto anual aproximado (antes de impuestos) de su hogar? Si su hijo comparte custodia en varios hogares, indique el ingreso total de todos esos hogares en conjunto.

- ○ Menos de $ 10,000 (1)
- ○ $ 10,000 - $ 19,999 (2)
- ○ $ 20,000 - $ 29,999 (3)
- ○ $ 30,000 - $ 39,999 (4)
- ○ $ 40,000 - $ 49,999 (5)
- ○ $ 50,000 - $ 59,999 (6)
- ○ $ 60,000 - $ 69,999 (7)
- ○ $ 70,000 - $ 79,999 (8)
- ○ $ 80,000 - $ 89,999 (9)
- ○ $ 90,000 - $ 99,999 (10)
- ○ $ 100,000 - $ 149,999 (11)
- ○ $ 150,000 - $ 199,999 (12)
- ○ $ 200,000 - $ 249,999 (13)
- ○ $ 250,000 - $ 299,999 (14)
- ○ $ 300.000 o más (15)
- ○ No lo sé (16)
- ○ Prefiero no contestar (17)

Q3.22 ¿Cuál es su lengua primaria?

- español (1)
- inglés (2)
- Otro idioma. Especifique: (3) ____

Q3.23 Identifique todos los idiomas que usted comprende con claridad (seleccione todos los que correspondan):

- español (1)
- inglés (2)
- Otro/s idioma/s. Especifique: (3) ____

Q3.24 Identifique todos los idiomas que usted habla con fluidez (seleccione todos los que correspondan):

- español (1)
- inglés (2)
- Otro/s idioma/s. Especifique: (3) ____

Q3.25 ¿Dónde habla usted español? (Seleccione todas las que correspondan):

- ◯ Hogar (1)
- ◯ Trabajo (2)
- ◯ Otro/s lugares/s. Especifique: (3) ____
- ◯ No hablo español en ninguna parte (4)

Q3.26 ¿Con quién habla usted en español? (Seleccione todas las que correspondan):

- ◯ Con otros adultos en casa (1)
- ◯ Con niños en casa (2)
- ◯ Con amigos y familiares (3)
- ◯ Con compañeros de trabajo (4)
- ◯ Con otra/s persona/s. Especifique: (5) ____
- ◯ Con nadie (6)

Q3.27 ¿Dónde habla usted inglés? (Seleccione todas las que correspondan):

- ◯ Hogar (1)
- ◯ Trabajo (2)
- ◯ Otro/s lugares/s. Especifique: (3) ____
- ◯ No hablo inglés en ninguna parte (4)

Q3.28 ¿Con quién habla usted en inglés? (Seleccione todas las que correspondan):

- ☐ Con otros adultos en casa (1)
- ☐ Con niños en casa (2)
- ☐ Con amigos y familiares (3)
- ☐ Con compañeros de trabajo (4)
- ☐ Con otra/s persona/s. Especifique: (5) ____
- ☐ Con nadie (6)

Q3.29 Ahora le haremos algunas preguntas sobre su/s hijo/s:

Q3.30 ¿Cuántos hijos tiene usted?

- ○ 1 (1)
- ○ 2 (2)
- ○ 3 (3)
- ○ 4 o más (4)

Q3.31 Si tiene mas de un hijo, centrémonos en uno de sus hijos.

- ○ ¿Cómo llama al niño/a sobre el que elige responder a las siguientes preguntas? (1) ____

Q3.32 Escriba cuántos años tiene ${Q3.31/ChoiceTextEntryValue/1} ?

____

Q3.33 Cuál es el género de ${Q3.31/ChoiceTextEntryValue/1} ?

- ○ Masculino (1)
- ○ Femenino (2)
- ○ Otro (especificar): (3) ____
- ○ Prefiero no contestar (4)

Q3.34 Identifique todos los idiomas que entiende ${Q3.31/ChoiceTextEntryValue/1}(seleccione todos los que correspondan):

- ▢ Español (1)
- ▢ Inglés (2)
- ▢ Otro (Especifique) (3) ____

Q3.35 Identifique todos los idiomas que habla ${Q3.31/ChoiceTextEntryValue/1}(seleccione todos los que correspondan):

- ▢ Español (1)
- ▢ Inglés (2)
- ▢ Otro (Especifique) (3) ____

Skip To: Q3.36 If Condition: Selected Count Is Greater Than or Equal to 2. Skip To: Indique si cree que ${q://QID45/Choic....

Skip To: Q3.38 If Condition: Selected Count Is Equal to 1. Skip To: Por favor, identifique todos los idio....

Q3.36 Indique si cree que ${Q3.31/ChoiceTextEntryValue/1} tiene preferencia por uno de los idiomas que habla:

- ☐ Sin preferencias habla los dos idiomas por igual (1)
- ☐ Tiene preferencia por el español (2)
- ☐ Tiene preferencia por el inglés (3)
- ☐ Tiene preferencia por otro idioma (Especifique) (4) ____

Display This Question:

If Indique si cree que ${q://QID144/ChoiceTextEntryValue/1} tiene preferencia por uno de los idiomas... , Sin preferencias habla los dos idiomas por igual Is Displayed

Q3.37 ¿Alguna vez ${Q3.31/ChoiceTextEntryValue/1} utiliza dos idiomas en la misma oración cuando habla?

- ○ Sí (1)
- ○ No (2)

Q3.38 Por favor, identifique todos los idiomas a los que ${Q3.31/ChoiceTextEntryValue/1} está expuesto en casa (directa o indirectamente). Seleccione todos los que correspondan:

- ☐ Español (1)
- ☐ Inglés (2)
- ☐ Otro idioma (especifique) (3) ____

Q3.39 Por favor, identifique en qué idioma o idiomas se comunica usted con ${Q3.31/ChoiceTextEntryValue/1}. (Seleccione todos los que correspondan)

☐ Español (1)

☐ Inglés (2)

☐ Otros idioma. Especifique (3) ____

Q3.40 ¿Quién le habla en cada idioma a ${Q3.31/ChoiceTextEntryValue/1} en el hogar (mamá, papá, hermanos,etc.)? (Escriba que personas tras todos los idiomas que correspondan)

○ En el hogar, a ${Q3.31/ChoiceTextEntryValue/1} le habla en español: (1) ____

○ En el hogar, a ${Q3.31/ChoiceTextEntryValue/1} le habla en ingles: (2) ____

○ En el hogar, a ${Q3.31/ChoiceTextEntryValue/1} le habla en otro idioma distinto del ingles o el español: (3) ____

Q3.41 Aproximadamente, ¿cuántas horas al día escucha ${Q3.31/ChoiceTextEntryValue/1} cada idioma en casa?

	En el hogar, no lo escucha nunca (1)	En el hogar, lo escucha de 1 a 3 horas diarias (2)	En el hogar, lo escucha de 4 a 6 horas diarias (3)	En el hogar, lo escucha de 7 a 9 horas diarias (4)	En el hogar, lo escucha mas de 10 horas diarias (5)
Español (1)	○	○	○	○	○
Inglés (2)	○	○	○	○	○
Otro idioma (especifique cual) (3)	○	○	○	○	○

Q3.42 ¿Usted o alguno de los adultos en su casa usa dos idiomas en la misma oración al hablar?

○ Sí (1)

○ No (2)

Q3.43 Por favor, identifique todos los idiomas a los que ${Q3.31/ChoiceTextEntryValue/1} está expuesto fuera del hogar. Seleccione todos los que correspondan:

▢ Español (1)

▢ Inglés (2)

▢ Otro idioma (especifique) (3) ____

Q3.44 ¿Dónde está expuesto ${Q3.31/ChoiceTextEntryValue/1} fuera del hogar (ejemplos: parque, escuela, ...)? (Escriba los lugares/contextos tras todos los idiomas que correspondan)

○ ${Q3.31/ChoiceTextEntryValue/1} está expuesto al español en: (1) ____

○ ${Q3.31/ChoiceTextEntryValue/1}está expuesto al ingles en: (2) ____

○ ${Q3.31/ChoiceTextEntryValue/1} está expuesto a otro idioma distinto del ingles o el español en: (3) ____

Q3.45 ¿Quién le habla en cada idioma a ${Q3.31/ChoiceTextEntryValue/1} fuera del hogar (ejemplos: amigos, vecinos, maestros, terapeutas...)? (Escriba que personas tras todos los idiomas que correspondan)

○ Fuera del hogar, a ${Q3.31/ChoiceTextEntryValue/1} le habla en español: (1) ____

○ Fuera del hogar, a ${Q3.31/ChoiceTextEntryValue/1} le habla en ingles: (2) ____

○ Fuera del hogar, a ${Q3.31/ChoiceTextEntryValue/1} le habla en otro idioma distinto del ingles o el español: (3) ____

Q3.46 Aproximadamente, ¿cuántas horas al día escucha ${Q3.31/ChoiceTextEntryValue/1} cada idioma fuera de casa? (Seleccione solo los idiomas que correspondan. Deje los demás en blanco.

	Fuera del hogar, no lo escucha nunca (1)	Fuera del hogar, lo escucha de 1 a 3 horas diarias (2)	Fuera del hogar, lo escucha de 4 a 6 horas diarias (3)	Fuera del hogar, lo escucha de 7 a 9 horas diarias (4)	Fuera del hogar, lo escucha de 10 a 16 horas diarias (5)
Español (1)	o	o	o	o	o
Inglés (2)	o	o	o	o	o
Otro idioma (especifique cual) (3)	o	o	o	o	o

Q3.47 ¿Cómo de bien cree que ${Q3.31/ChoiceTextEntryValue/1} entiende cada idioma? (Deje en blanco las opciones que no apliquen)

	Igual o mejor que los compañeros de su edad (1)	Algo peor que los compañeros de su edad (2)	Mucho peor que los compañeros de su edad (3)	No lo entiende en absoluto (4)
Español (1)	o	o	o	o
Inglés (2)	o	o	o	o
Otro idioma (especifique cual) (3)	o	o	o	o

Q3.48 ¿Cómo de bien cree que ${Q3.31/ChoiceTextEntryValue/1} habla cada idioma? (Deje en blanco las opciones que no apliquen)

	Igual o mejor que los compañeros de su edad (1)	Algo peor que los compañeros de su edad (2)	Mucho peor que los compañeros de su edad (3)	No lo habla en absoluto (4)
Español (1)	○	○	○	○
Inglés (2)	○	○	○	○
Otro idioma (especifique cual) (3)	○	○	○	○

Q3.49 ¿Cómo de bien cree que ${Q3.31/ChoiceTextEntryValue/1} lee en cada idioma? (Deje en blanco las opciones que no apliquen)

	Igual o mejor que los compañeros de su edad (1)	Algo peor que los compañeros de su edad (2)	Mucho peor que los compañeros de su edad (3)	No lo lee en absoluto (4)
Español (1)	○	○	○	○
Inglés (2)	○	○	○	○
Otro idioma (especifique cual) (3)	○	○	○	○

Q3.50 ¿Cómo de bien cree que ${Q3.31/ChoiceTextEntryValue/1} escribe en cada idioma? (Deje en blanco las opciones que no apliquen)

	Igual o mejor que los compañeros de su edad (1)	Algo peor que los compañeros de su edad (2)	Mucho peor que los compañeros de su edad (3)	No lo escribe en absoluto (4)
Español (1)	○	○	○	○
Inglés (2)	○	○	○	○
Otro idioma (especifique cual) (3)	○	○	○	○

Q3.51 Ahora le haremos algunas preguntas sobre su opinión como madre/padre sobre el bilingüismo:

Q3.52 ¿Cómo de importante es o fue para usted que ${Q3.31/ChoiceTextEntryValue/1} sea o fuese bilingüe?

- ○ extremadamente importante (1)
- ○ muy importante (2)
- ○ importante (3)
- ○ algo importante (4)
- ○ nada importante (5)

Display This Question:

If ¿Cómo de importante es o fue para usted que ${q://QID144/ChoiceTextEntryValue/1} sea o fuese bilin... != nada importante

Q3.53 Ordene de mas a menos importante las razones por las que cree que el bilingüismo es una meta importante para ${Q3.31/ChoiceTextEntryValue/1}

______ Porque quiero mantener nuestra cultura hispana (1)
______ Para que ${Q3.31/ChoiceTextEntryValue/1} se comunique con miembros de la familia que no hablan ingles (2)
______ Para que ${Q3.31/ChoiceTextEntryValue/1} se comunique con diversos miembros de la comunidad/vecindario (3)
______ Para que ${Q3.31/ChoiceTextEntryValue/1} se comunique con la gente de la escuela (4)
______ Vivo en una ciudad bilingüe / multilingüe (5)
______ Vivo en un pais bilingüe / multilingüe (6)
______ Brinda más oportunidades de vida (7)
______ Importante en el mercado laboral (8)
______ Otras razones. Por favor, especifique cuales: (9)

Q3.54 ¿Alguna vez pensó que hay motivos para que ${Q3.31/ChoiceTextEntryValue/1} no sea bilingüe?

◯ Sí, alguna vez pensé que hay razones para que ${Q3.31/ChoiceTextEntryValue/1} no sea bilingüe. Como por ejemplo: (1) ____

◯ No, jamas pensé que haya razones para que ${Q3.31/ChoiceTextEntryValue/1} no sea bilingüe (2)

Display This Question:

If ¿Alguna vez pensó que hay motivos para que ${q://QID144/ChoiceTextEntryValue/1} no sea bilingüe? = Sí, alguna vez pensé que hay razones para que ${q://QID144/ChoiceTextEntryValue/1} no sea bilingüe. Como por ejemplo:

Q3.55 A continuación ordene de más a menos preocupante los 5 principales motivos por los que usted pensó alguna vez que mejor que ${Q3.31/ChoiceTextEntryValue/1} no sea bilingüe.

______ Aprender más de un idioma es demasiado difícil para ${Q3.31/ChoiceTextEntryValue/1}. (1)
______ Hay poca o ninguna ayuda profesional para que ${Q3.31/ChoiceTextEntryValue/1} aprenda los dos idiomas. (2)
______ Un profesional (maestro / SLP / psicólogo / médico) me dijo que sería malo para ${Q3.31/ChoiceTextEntryValue/1} (3)
______ No puedo ayudar a ${Q3.31/ChoiceTextEntryValue/1} a aprender otro idioma. (4)
______ Tengo miedo de que ${Q3.31/ChoiceTextEntryValue/1} se confunda con dos idiomas. (5)
______ No tengo acceso a servicios que ayuden a ${Q3.31/ChoiceTextEntryValue/1} con el bilingüismo. (6)
______ Mi familia y / o amigos NO quieren ${Q3.31/ChoiceTextEntryValue/1} sea bilingüe (7)
______ Otros motivos (especificar) (8)

Q3.56 ¿Actualmente piensa que hay motivos para que ${Q3.31/ChoiceTextEntryValue/1} no sea bilingüe?

○ Sí, actualmente pienso que hayan motivos para que ${Q3.31/ChoiceTextEntryValue/1} no sea bilingüe. Como por ejemplo: (1) ____

○ No, actualmente no pienso que hayan motivos para que ${Q3.31/ChoiceTextEntryValue/1} no sea bilingüe (2)

Display This Question:

If ¿Actualmente piensa que hay motivos para que ${q://QID144/ChoiceTextEntryValue/1} no sea bilingüe? = Sí, actualmente pienso que hayan motivos para que ${q://QID144/ChoiceTextEntryValue/1} no sea bilingüe. Como por ejemplo:

Q3.57 A continuación ordene de más a menos preocupante los 5 principales motivos por los que actualmente piensa que mejor que ${Q3.31/ChoiceTextEntryValue/1} no sea bilingüe.

______ Aprender más de un idioma es demasiado difícil para ${Q3.31/ChoiceTextEntryValue/1}. (1)
______ Hay poca o ninguna ayuda profesional para que ${Q3.31/ChoiceTextEntryValue/1} aprenda los dos idiomas. (2)
______ Un profesional (maestro / SLP / psicólogo / médico) me dijo que sería malo para ${Q3.31/ChoiceTextEntryValue/1} (3)
______ No puedo ayudar a ${Q3.31/ChoiceTextEntryValue/1} a aprender otro idioma. (4)
______ Tengo miedo de que ${Q3.31/ChoiceTextEntryValue/1} se confunda con dos idiomas. (5)
______ No tengo acceso a servicios que ayuden a ${Q3.31/ChoiceTextEntryValue/1} con el bilingüismo. (6)
______ Mi familia y / o amigos NO quieren ${Q3.31/ChoiceTextEntryValue/1} sea bilingüe (7)
______ Otros motivos (especificar) (8)

Q3.58 ¿Qué elección ha hecho para ${Q3.31/ChoiceTextEntryValue/1}?

○ Decidí criar a ${Q3.31/ChoiceTextEntryValue/1} en una sola lengua (monolingüe). (1)

○ Decidí criar ${Q3.31/ChoiceTextEntryValue/1} en más de una lengua (bilingüe o multilingüe). (2)

Q3.59 ¿Cómo le hace sentir a usted esa elección?

○ Infeliz (1)

○ Ni feliz ni infeliz (2)

○ Feliz (3)

○ Muy feliz (4)

Q3.60 ¿Se arrepiente de la elección que tomó para ${Q3.31/ChoiceTextEntryValue/1}?

- ◯ Sí, me arrepiento. Por qué: (1) ____

- ◯ No me arrepiento. (2)

Display This Question:

If ¿Qué elección ha hecho para ${q://QID144/ChoiceTextEntryValue/1}? = Decidí criar a ${q://QID144/ChoiceTextEntryValue/1} en una sola lengua (monolingüe).

Q3.61 ¿En qué lengua decidió criar a ${Q3.31/ChoiceTextEntryValue/1}?

- ◯ Español (1)

- ◯ Inglés (2)

- ◯ Otra. Por favor especifique cual (3) ____

Display This Question:

If ¿Qué elección ha hecho para ${q://QID144/ChoiceTextEntryValue/1}? = Decidí criar ${q://QID144/ChoiceTextEntryValue/1} en más de una lengua (bilingüe o multilingüe).

Q3.62 Seleccione todas las estrategias que utiliza actualmente para ayudar a ${Q3.31/ChoiceTextEntryValue/1} a ser bilingüe:

- ☐ ${Q3.31/ChoiceTextEntryValue/1} toma clases de refuerzo para su segunda lengua (1)
- ☐ ${Q3.31/ChoiceTextEntryValue/1} recibe apoyo de un logopeda (SLP) bilingüe (2)
- ☐ Enseñamos a ${Q3.31/ChoiceTextEntryValue/1} habilidades lingüísticas en casa (3)
- ☐ Diferentes personas hablan diferentes idiomas en casa (4)
- ☐ Se hablan diferentes idiomas en diferentes momentos en casa (5)
- ☐ ${Q3.31/ChoiceTextEntryValue/1} asiste a la escuela en un idioma diferente al que hablamos predominantemente en casa (6)
- ☐ Ver televisión en dos (o más) idiomas (7)
- ☐ Leer libros en dos (o más) idiomas (8)
- ☐ Otro (especifique) (9) ____

Display This Question:

If ¿Qué elección ha hecho para ${q://QID144/ChoiceTextEntryValue/1}? = Decidí criar ${q://QID144/ChoiceTextEntryValue/1} en más de una lengua (bilingüe o multilingüe).

Q3.63 ¿Cómo calificaría su éxito en ayudar a ${Q3.31/ChoiceTextEntryValue/1} a ser bilingüe?

- ◯ no tuve éxito en absoluto (1)
- ◯ algo exitoso (2)
- ◯ exitoso (3)
- ◯ muy exitoso (4)
- ◯ extremadamente exitoso (5)

Display This Question:

If ¿Qué elección ha hecho para ${q://QID144/ChoiceTextEntryValue/1}? = Decidí criar ${q://QID144/ChoiceTextEntryValue/1} en más de una lengua (bilingüe o multilingüe).

Q3.64 Parece que usted eligió apoyar el bilingüismo de ${Q3.31/ChoiceTextEntryValue/1}, ¿qué tipo de ayuda ha resultado más útil para su hijo? Escriba en el cuadro de texto:

____

Display This Question:

If ¿Qué elección ha hecho para ${q://QID144/ChoiceTextEntryValue/1}? = Decidí criar a ${q://QID144/ChoiceTextEntryValue/1} en una sola lengua (monolingüe).

Q3.65 ¿Por qué decidió criar a ${Q3.31/ChoiceTextEntryValue/1} en una sola lengua (monolingüe)? Seleccione todas las que correspondan:

- ◯ Creo que aprender más de un idioma sería demasiado difícil para ${Q3.31/ChoiceTextEntryValue/1} . (1)
- ◯ Un profesional me dijo que aprender dos idiomas sería demasiado difícil para ${Q3.31/ChoiceTextEntryValue/1} (2)
- ◯ Hay poca o ninguna ayuda profesional para que ${Q3.31/ChoiceTextEntryValue/1} hable los dos idiomas (3)
- ◯ No puedo ayudar a ${Q3.31/ChoiceTextEntryValue/1} a aprender dos idioma (4)
- ◯ Tengo miedo de que ${Q3.31/ChoiceTextEntryValue/1} se confunda con dos idiomas (5)
- ◯ No tengo acceso a servicios que ayuden a ${Q3.31/ChoiceTextEntryValue/1} a ser bilingüe. (6)
- ◯ Mi familia y / o amigos no apoyarán que ${Q3.31/ChoiceTextEntryValue/1} aprenda otro idioma (7)
- ◯ Otro (especifique) (8) ____

Q3.66 Los profesionales a veces expresan opiniones sobre el bilingüismo. Seleccione qué consejo le ha dado cada profesional en relación con ${Q3.31/ChoiceTextEntryValue/1}. Si un profesional no le ha asesorado en esta área, déjelo en blanco.

- ☐ **Médico de familia (primary care phsyician PCP) (Seleccione si aplica):** (1)
- ☐ No críe a su hijo en más de una lengua. (Indique que motivos le dio el profesional para criar a su hijo de manera monolingüe): (2) ____
- ☐ Críe a su hijo en más de una lengua (bilingüe) (3)
- ☐ **Logopeda o terapista del habla y el lenguaje (Speech language pathologist (SLP):** (4)
- ☐ No críe a su hijo en más de una lengua. (Indique que motivos le dio el profesional para criar a su hijo de manera monolingüe): (5) ____
- ☐ Críe a su hijo en más de una lengua (bilingüe) (6)
- ☐ **Psicólogo:** (7)
- ☐ No críe a su hijo en más de una lengua. (Indique que motivos le dio el profesional para criar a su hijo de manera monolingüe): (8) ____
- ☐ Críe a su hijo en más de una lengua (bilingüe) (9)
- ☐ **Trabajador social:** (10)
- ☐ No críe a su hijo en más de una lengua. (Indique que motivos le dio el profesional para criar a su hijo de manera monolingüe): (11) ____
- ☐ Críe a su hijo en más de una lengua (bilingüe) (12)
- ☐ **Terapeuta de la conducta:** (13)

- ☐ No críe a su hijo en más de una lengua. (Indique que motivos le dio el profesional para criar a su hijo de manera monolingüe): (14) ____
- ☐ Críe a su hijo en más de una lengua (bilingüe) (15)
- ☐ **Maestros de la escuela:** (16)
- ☐ No críe a su hijo en más de una lengua. (Indique que motivos le dio el profesional para criar a su hijo de manera monolingüe): (17) ____
- ☐ Críe a su hijo en más de una lengua (bilingüe) (18)
- ☐ **Otro profesional. Especifique cual:** (19) ____
- ☐ No críe a su hijo en más de una lengua. (Indique que motivos le dio el profesional para criar a su hijo de manera monolingüe): (20) ____
- ☐ Críe a su hijo en más de una lengua (bilingüe) (21)

Q3.67 ¿Se siente apoyado por su comunidad para criar de manera bilingüe (en más de una lengua) a ${Q3.31/ChoiceTextEntryValue/1}?

- ○ Sí (1)
- ○ No (2)

Q3.68 ¿Cree que el bilingüismo se considera algo bueno en su comunidad?

- ○ Sí (1)
- ○ No (2)

Q3.69 ¿Alguna vez le ha dicho ${Q3.31/ChoiceTextEntryValue/1} que no quiere aprender ni hablar español?

- ◯ Si. Indique las razones que le dio ${Q3.31/ChoiceTextEntryValue/1} : (1) ____

- ◯ No, ${Q3.31/ChoiceTextEntryValue/1} nunca me dijo eso (2)

Q3.70 ¿Qué consejo le daría usted a otros padres que están pensando en criar a sus hijos de manera bilingüe o multilingüe en los Estados Unidos?

____

Q3.71 ¿Qué recursos recomendaría a otros padres que están pensando en criar a sus hijos de manera bilingüe o multilingüe en los Estados Unidos?

____

Q3.72 ¡Muchas gracias por participar!
